# Quantitative correlation between promoter methylation and messenger RNA levels of the reduced folate carrier

**DOI:** 10.1186/1471-2407-8-124

**Published:** 2008-05-01

**Authors:** Rui Yang, Wei-Wei Li, Bang H Hoang, Hansoo Kim, Debabrata Banerjee, Albert Kheradpour, John H Healey, Paul A Meyers, Joseph R Bertino, Richard Gorlick

**Affiliations:** 1 Department of Pediatrics and Molecular Pharmacology (R.Y., R.G.), The Albert Einstein College of Medicine, The Children's Hospital at Montefiore, Bronx, NY 10461, USA; 2 Department of Molecular Pharmacology and Therapeutics Program, Memorial Sloan-Kettering Cancer Center, New York, NY 10021, USA; 3 Orthopedic Surgery Service, Memorial Sloan-Kettering Cancer Center, New York, NY 10021, USA; 4 Department of Pediatrics (P.A.M.), Memorial Sloan-Kettering Cancer Center, New York, NY 10021, USA; 5 Department of Orthopaedic Surgery, University of California, Irvine, CA 92868, USA; 6 Department of Orthopaedic Surgery, Seoul National University Hospital, Chongno-gu, Seoul 110-744, Korea; 7 The Cancer Institute of New Jersey and Departments of Medicine and Pharmacology, University of Medicine and Dentistry of New Jersey, New Brunswick, NJ 08901, USA; 8 Department of Pediatric Oncology & Hematology, Loma Linda University Medical Center, Loma Linda, CA 92354, USA

## Abstract

**Background:**

Methotrexate (MTX) uptake is mediated by the reduced folate carrier (RFC). Defective drug uptake in association with decreased RFC expression is a common mechanism of MTX resistance in many tumor types. Heavy promoter methylation was previously identified as a basis for the complete silencing of RFC in MDA-MB-231 breast cancer cells, its role and prevalence in RFC transcription regulation are, however, not widely studied.

**Methods:**

In the current study, RFC promoter methylation was assessed using methylation specific PCR in a panel of malignant cell lines (n = 8), including MDA-MB-231, and M805, a MTX resistant cell line directly established from the specimen of a patient with malignant fibrohistocytoma, whom received multiple doses of MTX. A quantitative approach of real-time PCR for measuring the extent of RFC promoter methylation was developed, and was validated by direct bisulfite genomic sequencing. RFC mRNA levels were determined by quantitative real-time RT-PCR and were related to the extent of promoter methylation in these cell lines.

**Results:**

A partial promoter methylation and RFC mRNA down-regulation were observed in M805. Using the quantitative approach, a reverse correlation (correlation coefficient = -0.59, *p *< 0.05) was identified between the promoter methylation and RFC mRNA levels in this a panel of malignant cell lines.

**Conclusion:**

This study further suggests that promoter methylation is a potential basis for MTX resistance. The quantitative correlation identified in this study implies that promoter methylation is possibly a mechanism involved in the fine regulation of RFC transcription.

## Background

Methotrexate (MTX) remains an important drug in the treatment of a variety of malignancies, such as acute lymphocytic leukemia (ALL), choriocarcinoma, non-Hodgkin's lymphoma, osteosarcoma, breast cancer, and head and neck cancer. The mechanisms of MTX resistance include: 1) impaired drug transport; 2) reduced drug accumulation because of decreased MTX polyglutamylation or increased drug hydrolysis; 3) increased drug efflux possibly mediated by multiple drug resistance associated proteins (MRPs); 4) alterations in the structure or expression of the target enzyme dihydrofolate reductase (DHFR) and some novel mechanisms proposed recently [1-3]. MTX is delivered into cells predominantly via the reduced folate carrier (RFC), a bi-directional anion exchanger with 12 putative transmembrane domains [4]. To generate sufficient intracellular MTX, RFC transport of MTX is critical to its efficacy. Since the human RFC cDNA was cloned in 1995 [5-8], intensive efforts have been made to explore its clinical relevance in different tumor models. MTX resistance has been associated with decreased expression of RFC or loss of function of the carrier in many tumor types [9-12]. The RFC is ubiquitously expressed in normal tissues [13]; its regulatory mechanisms are, however, not clearly understood. Transcription of the RFC starts from at least four distinct promoters, (designated A, B, C, and D) [14], and is complicated by multiple 5'-non-coding exons resulting from alternative splicing [13,15-20]. The RFC promoter B appeared to be most potent in activity, and was predominantly utilized in tumor cells [16,19]. At least 18 different RFC transcripts have been reported, the functions of which are not clear, although links to tissue specific expression were suggested [13]. Further information on RFC regulation is of importance for understanding the mechanisms of MTX resistance in these diseases.

DNA methylation plays an important role in embryonic development and gene imprinting [21]. In cancer cells, DNA methylation in the promoter region is often involved in gene silencing, particularly for some tumor suppressor genes [22]. Recently, it was reported that a ~1400 bp region, including RFC promoter B and A, was identified as a CpG island [23]. Moreover, heavy promoter methylation was the underlying mechanism for the complete lack of RFC expression in MDA-MB-231 breast cancer cell line [24], associated with MTX resistance [23]. However, the role and prevalence of promoter methylation in RFC are not widely studied. A study was therefore initiated to further investigate the role of promoter methylation in a panel of malignant cell lines.

## Methods

### Cell Culture

The M805 cell line was directly established from a patient with malignant fibrohistocytoma (MFH), who was treated at Memorial Sloan-Kettering Cancer Center (MSKCC) and received multiple courses of chemotherapy, including MTX [25]. The fibrosarcoma cell line, HT1080; breast cancer cell lines MDA-MB-231, MCF-7; and leukemia cell line CCRF-CEM were purchased from ATCC (Rockville, MD). The MTX resistant leukemia cell line CEM-T and HL60R have been described previously [26,27]. The MTX resistant cell line, M316, was established from a relapsed pre-B ALL patient at MSKCC. Cells were maintained as monolayer or suspension in MEM-α media, supplemented with 10% fetal calf serum (Life technologies, Bethesda, MD), 100 units/ml penicillin and 3 mg/ml streptomycin at 37°C in a humidified atmosphere with 5% CO_2_.

### MTX Uptake, Polyglutamylation and Cytotoxicity Assays

[3',5',7-^3^H] MTX was purchased from Moravek (Brea, CA). MTX polyglutamate standards containing one to five additional glutamate residues were obtained from B. Schircks Labs (Jona, Switzerland). MTX uptake and intracellular MTX polyglutamtes were measured as described previously [27]. Non-labeled MTX was purchased from Sigma (St. Louis, MO) and trimetrexate (TMTX) was obtained from Warner Lambert/Parke-Davis (Ann Arbor, MI). Cell growth inhibition studies with MTX and TMTX were performed as described previously [27]. The fibrosarcoma cell line, HT1080, was used as a control in the MTX transport assays and cytotoxicity assays.

### Analysis of hRFC Transcripts

Total RNA was isolated using UltraspecRNA reagent (Biotecx, Houston, TX) according to manufacturer's instructions and was reverse-transcribed using Superscript II RT (Invitrogen, Bethesda, MD). The level of RFC mRNA was measured as described previously using quantitative real-time RT-PCR [28]. For M805 cells, the RFC mRNA was measured before and after the treatment with 5-aza-2'-deoxycytidine (Sigma, St. Louis, MO) at a concentration of 1 μM for 4 days, respectively. To screen for mutations in the RFC, cDNA from the M805 cell line was PCR-amplified using four pairs of overlapping primers spanning the entire coding region of RFC as described previously [29]. PCR products were gel-purified and subjected to automated sequencing on an ABI3100 sequencer (Applied Biosystems, Foster City, CA), or manual sequencing with a T7 Sequenase version 2.0 sequencing kit (Amersham, Piscataway, NJ) in both directions.

### Transfection of hRFC cDNA

The transfection of the full-length human RFC cDNA into the RFC null, MDA-MB-231 cells was carried out with a SuperFect Transfection Reagent Kit (Qiagen, Valencia CA) according to manufacturer's instructions as described previously [30]. Stable transfection was obtained by selection with zeocin (Invitrogen, Carlsbad, CA) at a concentration of 400 μg for 2 weeks. Zeocin resistant colonies were obtained and were further analyzed for RFC expression by real-time RT-PCR.

### Methylation Specific PCR (MSP)

Genomic DNA was extracted using a QIAamp DNeasy Tissue Kit (Qiagen, Valencia, CA) according to manufacturer's instructions.

Bisulfite treatment was carried out as described previously [23]. Modified DNA was PCR-amplified with two pairs of primers targeting the same region (close to promoter B) of 110 base pair in length, but specific for the methylated RFC promoter and the unmethylated promoter respectively as described previously [23]. Primers specific to the RFC promoter A were designed as shown in Table 1. The annealing temperature was 58°C for methylated promoter A and 54°C for the unmethylated one. PCR products of 132 bp or 134 bp in length were separated on 2% agarose gels with ethidium bromide visualization under UV light. The breast cancer cell lines, MDA-MB-231 and MCF-7 were used as controls for methylated RFC promoter and unmethylated RFC promoter respectively as described previously [23].

**Table 1:** Primers used in methylation specific PCR and bisulfite genomic sequnecing

**Methylation Specific PCR (MSP)**	Annealing	position^a^
Promoter A (methylated)	58°C	1711–1842
Forward: 5'-TTC GTC GTA GTT TGC GAA TG		1711–1730
Reverse: 5'-CAA CAC GTA CCT AAA CGC GA		1842-1821
Promoter A (unmethylated)	54°C	1710–1843
Forward: 5'-TTT GTT GTA GTT TGT GAA TGG		1710–1730
Reverse: 5'-ACA ACA CAT ACC TAA ACA CAA		1843-1823
Promoter B		Ref. [23]

**Real-time Quantitative MSP**		

Promoter B (Methylated)	60°C	1171–1293
Forward: 5'-TTG TCG TAG CGT TCG GTT AC		1171–1190
Reverse: 5'-AAA CTA CAA CGC CCA CAA AA		1293 – 1274
Probe: 5'-Fam-TCG CGG GAC GGA TTC GTT TA		1218–1237

**Bisulfite Genomic Sequencing**		

Promoter B	58°C	1141–1454
Forward: 5'-tgg gtg gga ggg tgt tt		1141–1157
Reverse: 5'-cct cac aaa acc cta caa acc t		1454-1433

a: Numbering is based on NCBI accession number U92868.

### Quantitative Real-time Methylation Specific PCR

Primers and fluorescent probes based on sodium bisulfite treatment were designed as shown in Table 1. The RFC promoter B (minimal sequences: nt.1233–1278) was flanked by the PCR primers [19]. Fluorescent real-time PCR was performed in a reaction volume of 50 μl using components of a Taqman PCR Buffer A Pack (PE Biosystems, Branchburg, NJ). Three microliters of treated genomic DNA were used in each reaction including 1 × Taqman buffer A with 600 nM each primer, 200 nM of probe, 200 μM of dNTP mix and 5.5 mM MgCl_2_. Thermal cycling was carried out on a Bio-Rad iCycler with a denaturation step of 95°C for 10 min, 50 cycles of 95°C for 15 s, 60°C for 1 min. Genomic DNA treated with SssI methyl transferase (New England Biolab, Beverly, MA) was used as a control for methylated promoter. MYOD1 was used as internal control because of the lack of CpG sites on the primers and probe as described previously [31]. Multiple wells of non-template control were included on each 96-well PCR plate. All the samples were tested in triplicate for the RFC and MYOD1 respectively.

### Bisulfite Genomic Sequencing

Bisulfite genomic sequencing was used to validate the results obtained by quantitative real-time MSP in M805, MDA-MB-231, and MCF-7 cells. Primers flanking RFC promoter B were designed in regions lacking CpG sites (Table 1), thus amplification (covering 46 CpG sites) was independent of methylation status. The 314 bp PCR product was separated onto a 1% agarose gel and was purified with a Gel purification Kit (Qiagen, Valencia, CA). The PCR product was cloned using a TOPO-PCR TA cloning vector (Invitrogen, Carlsbad, CA) and at least 10 random clones were sequenced on an ABI 3100 auto sequencer with M13 reverse and forward primers.

### Statistical analysis

Statistical analysis was performed using a software package SPCC10 (Chicago, IL). Pearson correlation was used to assess the association between the promoter methylation and RFC mRNA levels, and a *p *< 0.05 was regarded as statistically significant.

## Results

**Figure 1:**
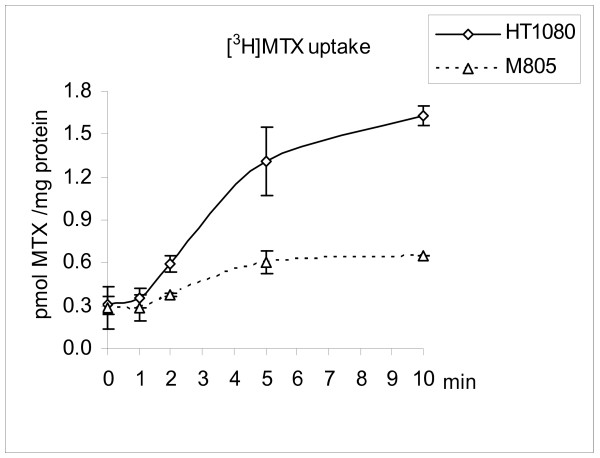
**[^3^H]-MTX uptake in M805 cells and HT1080 cells**. Cells were incubated with 1 μM [^3^H]-MTX and incubations were stopped at indicated time points. For each sample, experiments were performed in duplicates for each time point. Average value and standard error are shown.

The M805 cell line, obtained from a MFH patient treated with multiple courses of MTX, developed resistance to MTX *in vivo*[25]. A 15-fold resistance to MTX was observed in M805 cells (IC_50 _= 2.30 μM) as compared to a fibrosarcoma cell line, HT1080 (IC_50 _= 0.15 μM). In contrast, these two cell lines were almost equally sensitive to TMTX (IC_50_: 24 nM for M805 and 19 nM for HT1080, respectively), which enters cells through passive diffusion. This suggested that the MTX resistance might be the result of impaired drug uptake. MTX transport assay was performed as shown in Figure 1. A 3.0, 3.1, and 3.6-fold decrease of [^3^H]-MTX uptake in M805 cells was found at 2, 5, and 10 min respectively, as compared to the HT1080 cells. The intracellular concentrations of MTX and MTX polyglutamates after 24 h incubation were measured as shown in Table 2. Although the total (N = 1–5) MTX polyglutamates in M805 cells was only about 55% of that in HT1080 cells in consistent with defective drug transport, long-chain polyglutamates (N = 3–5) accounted for 54.8% of the polyglutamates in M805 cells as compared to 39% in HT1080 cells, suggesting that the M805 cell line did not have impaired MTX polyglutamylation. Using quantitative real-time RT-PCR, a 5-fold decrease in RFC mRNA expression was detected in M805 cells as compared to HT-1080, while there was no detectable RFC mRNA in MDA-MB-231 cells in consistent with previous report [23]. The down-regulation of RFC mRNA in M805 cell was not apparently changed after growing cells for 2 weeks in folate-free media (Life technologies, Bethesda, MD) supplemented with 20 nM leucovorin, in stead of standard media containing 2.3 μM folic acid. To screen for mutations in the M805 cells, the entire coding region of the RFC was sequenced, but no mutation was identified.

**Table 2:** Intracellular MTX Polyglutamates after 24 h incubation with 10 uM ^3^H-MTX

Cell lines	Quantities of MTX polyglutamates (pmol/10^7 ^cells)						
	
	N^a ^= 1	2	3	4	5	Total	Long chain (% of total) MTX(Glu)_3–5_
M805	60.4	7.9	25.0	38.2	19.5	151.0	82.7 (54.8%)
HT1080	135.0	33.0	54.0	37.0	15.0	274.0	106.0 (39%)

a: Indicates the number of additional glutamate residues.

**Figure 2:**
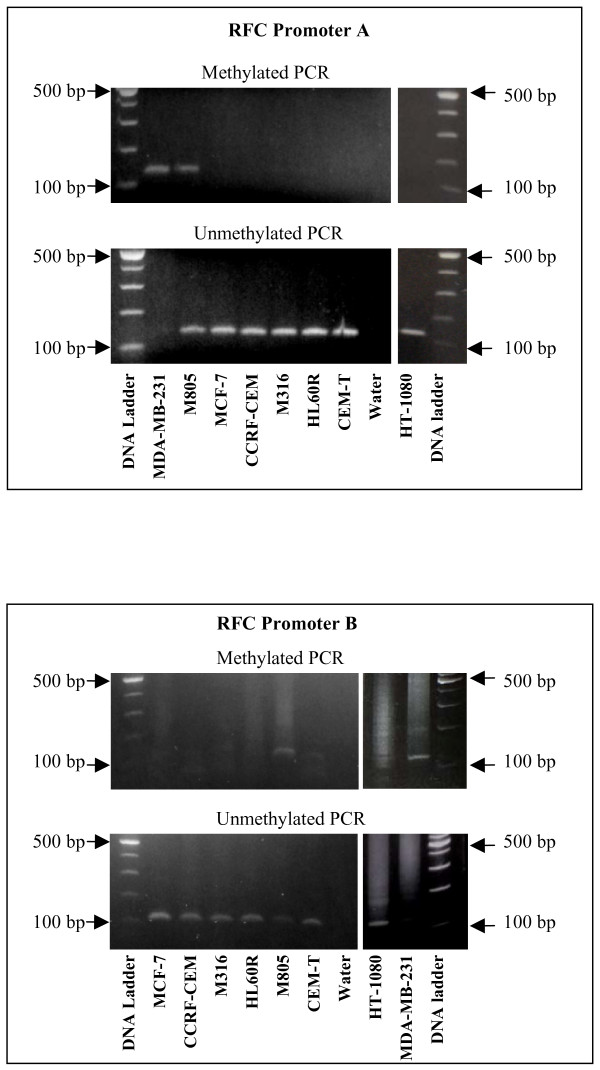
**Methylation specific PCR (MSP) on the RFC promoter**. Promoter methylation was assayed by MSP in a panel of malignant cell lines. Only in the breast cancer cell line MDA-MB-231 and the M805 cells, is there PCR-amplification with the primers specific for the methylated RFC promoter A and B, while there is amplification with the primers specific for the unmethylated promoters in all other cell lines but not MDA-MB-231 cells using the same templates. Water was used as a non-template control.

Heavy promoter methylation was identified to be the basis for complete silencing of RFC in MDA-MB-231 cells [23]. MSP was therefore performed in M805 cells in search for the factors responsible for its RFC down-regulation. As shown in Figure 2, PCR products in length of 132 bp for promoter A, and of 110 bp for promoter B were amplified in reactions with primers specific for methylated promoters in the MDA-MB-231 and M805 cells, but not in the other cell lines analyzed, including CCRF-CEM, CEM-T, HL60R, M316, MCF-7, and HT1080. Being different from the RFC-null MDA-MB-231, promoter methylation in RFC-low M805 cells was partial, because weak amplification was also observed in reactions with primers specific for the unmethylated promoters (Figure 2). After treatment with 5-aza-2'-deoxycytidine, RFC expression was increased by 2.9-fold in M805 cells, providing evidence that the partial promoter methylation is a mechanism for the RFC down-regulation in this cell line.

Stable transfection of human RFC cDNA into the RFC-null, MDA-MB-231 cells restored the sensitivity to MTX by more than 100 fold (data not shown). Transfection of RFC cDNA into M805 cells proved to be problematic after multiple attempts. After the treatment of 5-aza-2'-deoxycytidine, a marginal (1.4-fold) increase of sensitivity to MTX was observed in M805 cells as compared to the untreated cells. This was similarly seen in MDA-MB-231 cells, perhaps due to increased MRPs expression after the treatment as reported previously [23].

**Figure 3:**
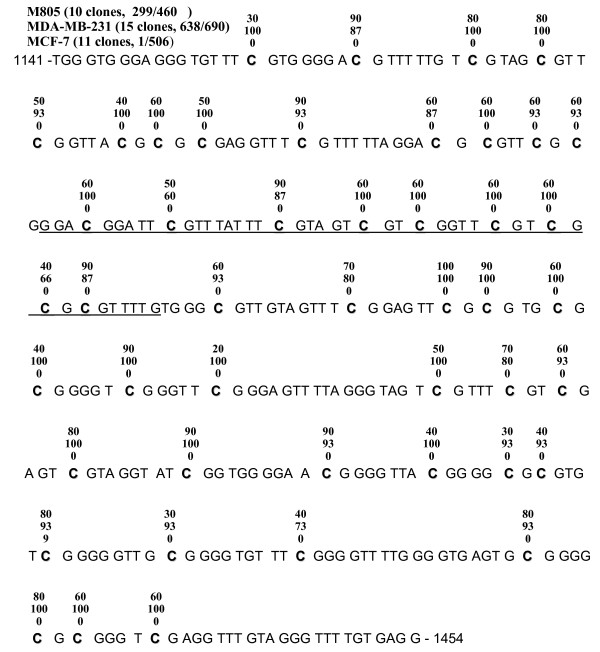
**Bisulfite Genomic Sequencing**. Bisulfite genomic sequencing was used to validate the methylation levels measured by real-time quantitative MSP in M805 cells, MDA-MB-231 cells and MCF-7 cells. Sequences of the PCR products (nucleotides no. 1141–1464, Access number: U92868) are mapped, as all the cytidines except those in CpG dinucleotides are converted to uracil (as thymidine in figure) after sodium bisulfite modification. The RFC promoter B is underlined. The number above each cytidine in CpG binucleotide (shown in bold) indicates the percentage of methylated cytidines on the respective site, respectively. The numbers in parenthesis indicate total clonies sequenced and total number of methylated cytidines in each cell line analyzed.

To further assess the relationship between the RFC mRNA expression and promoter methylation, a quantitative approach was adapted using the methodology developed by Sidransky et al. as described in the Methods [31]. The methylation level of the RFC promoter B was determined to be 50.6% for MDA-MB-231 cells, 21.8% for M805 cells, 0.3% for CCRF-CEM cells, and 0% for MCF-7 cells respectively, as compared to that of the DNA treated with Sss I methyltransferase *in vitro *(arbitrarily defined as 100% methylated). To validate these results, genomic DNA from MDA-MB-231, M805, and MCF-7 cells was further examined by bisulfite genomic sequencing. As shown in Figure 3, for MDA-MB-231, 638 out of total 690 (92%) CpG sites were methylated, and eight out of fifteen (53%) clones were expected to be amplified by real-time MSP. (CpG sites 3–5 are covered by sense primer, site 23 by antisense primer, and sites 12–15 by the fluorescent probe. When all these sites are simultaneously methylated, template will be amplified by real-time MSP.) For M805 cells, 299 out of total 460 (65%) CpG sites (10 clones) were methylated. Two out of ten (20%) clones sequenced for M805 cells were expected to be amplified by real-time MSP. For MCF-7 cells, only one out of 506 CpG sites was methylated. These results are in excellent agreement with the data obtained by real-time MSP.

**Figure 4:**
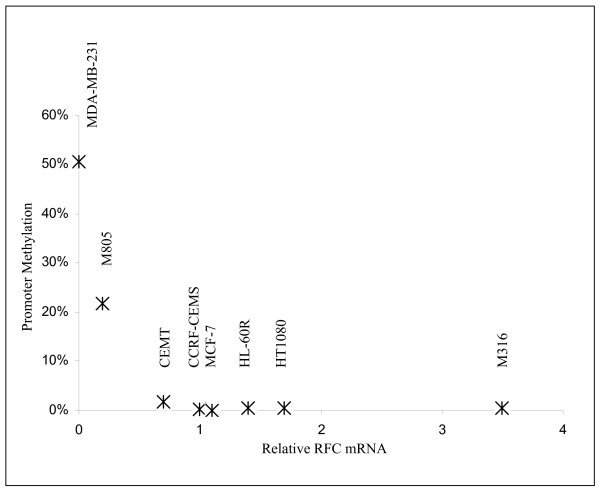
**Correlation between promoter methylation and RFC mRNA levels**. The relative level of RFC mRNA in each cell line is shown on the "x" axis as folds relative to that of CCRF-CEM (arbitrarily defined as 1), as measured by quantitative real-time RT-PCR. The extents of RFC promoter B methylation was shown on the "y" axis as percentage relative to that of SssI methyl transferase treated genomic DNA (arbitrarily defined as 100%), as measured by quantitative real-time MSP. The cell lines (n = 8) are indicated above each of the data points, respectively. A reverse correlation (Correlation coefficient = -0.59, *p *< 0.05) was obtained in this panel of cell lines.

This quantitative real-time MSP was further utilized in a panel of cell lines to determine the extents of promoter methylation and results are shown in Figure 4. The RFC mRNA levels were measured by real-time RT-PCR (Figure 4). When the extents of promoter methylation measured in this panel of cell lines were related to their RFC mRNA levels, a reverse correlation was obtained (Correlation coefficient = -0.59, *p *< 0.05). It revealed that high levels of promoter methylation were only seen in cell lines with decreased RFC mRNA expression, such as MDA-MB-231 and M805 cells, but not in any of the cell lines with higher RFC mRNA expression, including CCRF-CEM, HL60R, CEM-T, M316, MCF-7, and HT1080 cell lines (Figure 4).

## Discussion

Promoter methylation is associated with RFC down-regulation in the M805 cells, which resulted in impaired MTX uptake and drug resistance. Potential efflux mechanisms to explain decreased MTX uptake were partially excluded by the fact that MRP-1 and MRP-2 were not up-regulated in M805 cells as determined by quantitative real-time RT-PCR (data not shown). This is consistent with previous studies performed using immuno-histochemistry [25]. As reported in the prior study, mutations in the TP53 and Rb genes are not present in M805 cells, which exclude this as a possible mechanism of MTX resistance through alterations in cell cycle regulation [3]. The M805 cell line was resistant to MTX when established from a patient's specimen, who received pre-operative chemotherapy, including multiple doses of MTX. The breast cell line, MDA-MB-231, also developed MTX resistance *in vivo *due to the complete loss of RFC expression resulting from heavy promoter methylation [23,24]. This suggests that RFC promoter methylation may be a possible mechanism of MTX resistance developed by tumor cells *in vivo*. Defective MTX uptake mediated by the RFC is a common mechanism for drug resistance in many tissue types, including ALL, osteosarcoma, and non-Hodgkin's lymphoma [32]. The role of promoter methylation and prevalence of it is not yet widely investigated in these diseases. An obstacle of it is the current methodology. The regular MSP is sensitive; however, the result of it is only qualitative. The bisulfite genomic sequencing with the most accuracy is not a practical approach for patient samples. The quantitative real-time MSP developed in the current study appeared to be both sensitive and consistent with data obtained by bisulfite genomic sequencing. It is therefore reasonable to apply it in a bigger setting of patient samples in future studies.

The implications of multiple promoter usage and heterogeneity of RFC transcripts in normal and tumor tissue remain unclear. In this study, at least for MDA-MB-231 and M805 cell lines, DNA methylation occurred simultaneously at both promoter regions. Meanwhile, for the other cell lines included in this study with higher RFC mRNA expression, DNA methylation was not seen at either promoter region (Figure 2). Therefore, the pattern of promoter methylation in RFC promoter appears to be in a "both or neither" fashion, given the proximity in distance between these two promoters. Therefore, promoter methylation is unlikely involved in the selective usage of multiple promoters in the RFC transcription. However, the role of methylation is yet to be determined in relationship with transcriptional activities of other RFC promoters reported recently [14]. Furthermore, 5-aza-2'-deoxycytosine treatment only marginally reversed MTX resistance in M805 cells, or similarly in MDA-MB-231 cells [23]. This suggests that a more specific means of demethylation is perhaps needed to avoid the up-regulation of genes involved in the drug efflux system upon treatment.

The quantitative correlation between promoter methylation and RFC mRNA levels observed in this study is intriguing. This suggests that the extent of promoter methylation may directly reflect the promoter activity, perhaps is a fine mechanism in concert with other regulatory elements [14], as similarly observed in the tumor suppressor gene APC [31]. The patterns of CpG island methylation revealed by the bisulfite genomic sequencing in MDA-MB-231 and M805 cells (Figure 3) suggest that the overall extent of CpG island methylation is perhaps more important than that of specific sites. This is in contrast to the effect of genetic mutations in the promoter region, which usually abort the binding sites of transcription factors. This suggests that the overall compactness of DNA conferred by the promoter methylation is perhaps the determinant of transcription activity in the RFC. Although it is unknown whether methylation, or gene silencing, occurs first, DNA methylation is important for the maintenance of a silenced gene [22]. It is possible that the quantitative methylation is a means for cells to maintain the RFC mRNA transcription at a desired level, rather than in a "whole or none" fashion. A future study including a larger volume of samples is required to further determine the role of promoter methylation in the RFC transcription regulation.

## Conclusion

In this study, we have developed a quantitative approach measuring the extents of RFC promoter methylation. This study further suggests that promoter methylation is a potential basis for MTX resistance. The quantitative correlation identified in this panel of malignant cell lines implies that promoter methylation is a possible mechanism involved in the fine regulation of RFC transcription. Future study is necessary to clarify the role of promoter methylation in RFC transcription regulation.

## Abbreviations

RFC, reduced folate carrier; MTX, methotrexate; DHFR, dihydrofolate reductase; MSP, methylation specific PCR.

## Competing interests

The authors declare that they have no competing interests.

## Authors' contributions

RY carried out experiment design, molecular assays, and drafting the manuscript.

WWL carried out the MTX cytotoxicity, MTX transport, and polyglutamylation assays.

BHH and HK carried out real-time RT-PCR, data analysis, and statistical analysis.

DB, AK, and JRB participated in drafting and revising the manuscript.

JHH and PAM carried out data interpretation, and manuscript revision.

RG carried out the experiment design, data collection, manuscript drafting and revision.

## Pre-publication history

The pre-publication history for this paper can be accessed here:



## References

[B1] Zhao R, Goldman ID (2003). Resistance to antifolates. Oncogene.

[B2] Gorlick R, Goker E, Trippett T, Waltham M, Banerjee D, Bertino JR (1996). Intrinsic and acquired resistance to methotrexate in acute leukemia. N Engl J Med.

[B3] Banerjee D, Mayer-Kuckuk P, Capiaux G, Budak-Alpdogan T, Gorlick R, Bertino JR (2002). Novel aspects of resistance to drugs targeted to dihydrofolate reductase and thymidylate synthase. Biochim Biophys Acta.

[B4] Matherly LH, Goldman DI (2003). Membrane transport of folates. Vitam Horm.

[B5] Wong SC, Proefke SA, Bhushan A, Matherly LH (1995). Isolation of human cDNAs that restore methotrexate sensitivity and reduced folate carrier activity in methotrexate transport-defective Chinese hamster ovary cells. J Biol Chem.

[B6] Williams FM, Flintoff WF (1995). Isolation of a human cDNA that complements a mutant hamster cell defective in methotrexate uptake. J Biol Chem.

[B7] Prasad PD, Ramamoorthy S, Leibach FH, Ganapathy V (1995). Molecular cloning of the human placental folate transporter. Biochem Biophys Res Commun.

[B8] Moscow JA, Gong M, He R, Sgagias MK, Dixon KH, Anzick SL, Meltzer PS, Cowan KH (1995). Isolation of a gene encoding a human reduced folate carrier (RFC1) and analysis of its expression in transport-deficient, methotrexate-resistant human breast cancer cells. Cancer Res.

[B9] Zhang L, Taub JW, Williamson M, Wong SC, Hukku B, Pullen J, Ravindranath Y, Matherly LH (1998). Reduced folate carrier gene expression in childhood acute lymphoblastic leukemia: relationship to immunophenotype and ploidy. Clin Cancer Res.

[B10] Gorlick R, Goker E, Trippett T, Steinherz P, Elisseyeff Y, Mazumdar M, Flintoff WF, Bertino JR (1997). Defective transport is a common mechanism of acquired methotrexate resistance in acute lymphocytic leukemia and is associated with decreased reduced folate carrier expression. Blood.

[B11] Ma D, Huang H, Moscow JA (2000). Down-regulation of reduced folate carrier gene (RFC1) expression after exposure to methotrexate in ZR-75-1 breast cancer cells. Biochem Biophys Res Commun.

[B12] Guo W, Healey JH, Meyers PA, Ladanyi M, Huvos AG, Bertino JR, Gorlick R (1999). Mechanisms of methotrexate resistance in osteosarcoma. Clin Cancer Res.

[B13] Whetstine JR, Flatley RM, Matherly LH (2002). The human reduced folate carrier gene is ubiquitously and differentially expressed in normal human tissues: identification of seven non-coding exons and characterization of a novel promoter. Biochem J.

[B14] Matherly LH, Hou Z, Deng Y (2007). Human reduced folate carrier: translation of basic biology to cancer etiology and therapy. Cancer Metastasis Rev.

[B15] Zhang L, Wong SC, Matherly LH (1998). Transcript heterogeneity of the human reduced folate carrier results from the use of multiple promoters and variable splicing of alternative upstream exons. Biochem J.

[B16] Tolner B, Roy K, Sirotnak FM (1998). Structural analysis of the human RFC-1 gene encoding a folate transporter reveals multiple promoters and alternatively spliced transcripts with 5' end heterogeneity. Gene.

[B17] Williams FM, Flintoff WF (1998). Structural organization of the human reduced folate carrier gene: evidence for 5' heterogeneity in lymphoblast mRNA. Somat Cell Mol Genet.

[B18] Gong M, Cowan KH, Gudas J, Moscow JA (1999). Isolation and characterization of genomic sequences involved in the regulation of the human reduced folate carrier gene (RFC1). Gene.

[B19] Whetstine JR, Matherly LH (2001). The basal promoters for the human reduced folate carrier gene are regulated by a GC-box and a cAMP-response element/AP-1-like element. Basis for tissue-specific gene expression. J Biol Chem.

[B20] Whetstine JR, Witt TL, Matherly LH (2002). The human reduced folate carrier gene is regulated by the AP2 and sp1 transcription factor families and a functional 61-base pair polymorphism. J Biol Chem.

[B21] Bird A (2002). DNA methylation patterns and epigenetic memory. Genes Dev.

[B22] Jones PA, Baylin SB (2002). The fundamental role of epigenetic events in cancer. Nat Rev Genet.

[B23] Worm J, Kirkin AF, Dzhandzhugazyan KN, Guldberg P (2001). Methylation-dependent silencing of the reduced folate carrier gene in inherently methotrexate-resistant human breast cancer cells. J Biol Chem.

[B24] Moscow JA, Connolly T, Myers TG, Cheng CC, Paull K, Cowan KH (1997). Reduced folate carrier gene (RFC1) expression and anti-folate resistance in transfected and non-selected cell lines. Int J Cancer.

[B25] Li WW, Takahashi N, Jhanwar S, Cordon-Cardo C, Elisseyeff Y, Jimeno J, Faircloth G, Bertino JR (2001). Sensitivity of soft tissue sarcoma cell lines to chemotherapeutic agents: identification of ecteinascidin-743 as a potent cytotoxic agent. Clin Cancer Res.

[B26] Dedhar S, Hartley D, Goldie JH (1985). Increased dihydrofolate reductase activity in methotrexate-resistant human promyelocytic-leukaemia (HL-60) cells. Lack of correlation between increased activity and overproduction. Biochem J.

[B27] Mini E, Moroson BA, Franco CT, Bertino JR (1985). Cytotoxic effects of folate antagonists against methotrexate-resistant human leukemic lymphoblast CCRF-CEM cell lines. Cancer Res.

[B28] Sowers R, Toguchida J, Qin J, Meyers PA, Healey JH, Huvos A, Banerjee D, Bertino JR, Gorlick R (2003). mRNA expression levels of E2F transcription factors correlate with dihydrofolate reductase, reduced folate carrier, and thymidylate synthase mRNA expression in osteosarcoma. Mol Cancer Ther.

[B29] Wong SC, Zhang L, Witt TL, Proefke SA, Bhushan A, Matherly LH (1999). Impaired membrane transport in methotrexate-resistant CCRF-CEM cells involves early translation termination and increased turnover of a mutant reduced folate carrier. J Biol Chem.

[B30] Yang R, Kolb EA, Qin J, Chou A, Sowers R, Hoang B, Healey JH, Huvos AG, Meyers PA, Gorlick R (2007). The folate receptor alpha is frequently overexpressed in osteosarcoma samples and plays a role in the uptake of the physiologic substrate 5-methyltetrahydrofolate. Clin Cancer Res.

[B31] Usadel H, Brabender J, Danenberg KD, Jeronimo C, Harden S, Engles J, Danenberg PV, Yang S, Sidransky D (2002). Quantitative adenomatous polyposis coli promoter methylation analysis in tumor tissue, serum, and plasma DNA of patients with lung cancer. Cancer Res.

[B32] Ferreri AJ, Dell'Oro S, Capello D, Ponzoni M, Iuzzolino P, Rossi D, Pasini F, Ambrosetti A, Orvieto E, Ferrarese F, Arrigoni G, Foppoli M, Reni M, Gaidano G (2004). Aberrant methylation in the promoter region of the reduced folate carrier gene is a potential mechanism of resistance to methotrexate in primary central nervous system lymphomas. Br J Haematol.

